# Eicosanoid Profiling in an Orthotopic Model of Lung Cancer Progression by Mass Spectrometry Demonstrates Selective Production of Leukotrienes by Inflammatory Cells of the Microenvironment

**DOI:** 10.1371/journal.pone.0079633

**Published:** 2013-11-11

**Authors:** Joanna M. Poczobutt, Miguel Gijon, Jay Amin, Dwight Hanson, Howard Li, Deandra Walker, Mary Weiser-Evans, Xian Lu, Robert C. Murphy, Raphael A. Nemenoff

**Affiliations:** 1 Department of Medicine, University of Colorado Denver, Aurora, Colorado, United States of America; 2 Department of Pharmacology, University of Colorado Denver, Aurora, Colorado, United States of America; 3 Department of Biostatistics and Informatics, Colorado School of Public Health, University of Colorado Denver, Aurora, Colorado, United States of America; 4 Veterans Affairs Medical Center, Denver, Colorado, United States of America; University of Southampton School of Medicine, United Kingdom

## Abstract

Eicosanoids are bioactive lipid mediators derived from arachidonic acid^1^ (AA), which is released by cytosolic phospholipase A_2_ (cPLA_2_). AA is metabolized through three major pathways, cyclooxygenase (COX), lipoxygenase (LO) and cytochrome P450, to produce a family of eicosanoids, which individually have been shown to have pro- or anti-tumorigenic activities in cancer. However, cancer progression likely depends on complex changes in multiple eicosanoids produced by cancer cells and by tumor microenvironment and a systematic examination of the spectrum of eicosanoids in cancer has not been performed. We used liquid chromatography coupled with tandem mass spectrometry (LC/MS/MS) to quantitate eicosanoids produced during lung tumor progression in an orthotopic immunocompetent mouse model of lung cancer, in which Lewis lung carcinoma (LLC) cells are injected into lungs of syngeneic mice. The presence of tumor increased products of both the cyclooxygenase and the lipoxygenase pathways in a time-dependent fashion. Comparing tumors grown in cPLA_2_ knockout vs wild-type mice, we demonstrated that prostaglandins (PGE_2_, PGD_2_ and PGF_2a_) were produced by both cancer cells and the tumor microenvironment (TME), but leukotriene (LTB_4_, LTC_4_, LTD_4_, LTE_4_) production required cPLA_2_ expression in the TME. Using flow cytometry, we recovered tumor-associated neutrophils and 2 types of tumor-associated macrophages from tumor-bearing lungs and we defined their distinct eicosanoid profiles by LC/MS/MS. The combination of flow cytometry and LC/MS/MS unravels the complexity of eicosanoid production in lung cancer and provides a rationale to develop therapeutic strategies that target select cell populations to inhibit specific classes of eicosanoids.

## Introduction

Eicosanoids represent a family of bioactive lipids produced through metabolism of arachidonic acid. Arachidonic acid (AA) is a polyunsaturated fatty acid, which is incorporated into the sn-2 position of membrane phospholipids. The family of PLA_2_ enzymes hydrolyze membrane phospholipids to produce free fatty acids and lysophospholipids. While multiple forms of PLA_2_ have been identified, cytosolic PLA_2_-α, designated in this study as cPLA_2_, is specific for arachidonoyl-containing phospholipids, and is the major enzyme involved in regulated release of AA in response to mitogenic or inflammatory stimuli [1]. Free AA can be metabolized through three major pathways [2]. Cyclooxygenases (COX-1, 2) produce prostaglandins, including PGE_2_ and PGI_2_ as well as thromboxanes, lipoxygenases produce hydroxyeicosatetraenoic acids (HETEs) and leukotrienes, and cytochrome P450 epoxygenases produce epoxygenated fatty acids (EETs). Over 100 distinct eicosanoid species have been identified [3]. The majority of these molecules are secreted from cells and act in an autocrine or paracrine fashion through a family of G-protein coupled receptors [4]. The repertoire of eicosanoids produced by a particular cell type will be governed by expression of enzymes in the pathway, including specific downstream synthases. For example, PGE_2_ production will be regulated by expression of cyclooxygenase enzymes as well as prostaglandin E2 synthases, while specific leukotrienes, such as LTC_4_ require expression of 5-lipoxygenase, as well as LTC_4_ synthase.

Lung cancer is associated with the highest number of cancer deaths in both men and women, underscoring the need for novel therapeutic and preventive approaches [5]. Studies in a variety of cancers, including lung cancer, have implicated individual eicosanoids as mediators of cancer initiation, progression and metastasis. Most extensively studied are prostaglandins, specifically PGE_2._ Studies in cancer cell lines have demonstrated increased production of PGE_2_ mediated through induction of COX-2 and cPLA_2_ expression [6]–[8]. Inhibition of prostaglandin production via blocking either cPLA_2_ or COX-2 inhibits transformed growth of non-small cell lung cancer cells (NSCLC), and the development of tumors in mice in response to chemical carcinogens [9]. We demonstrated that mice that are deficient in cPLA_2_ (cPLA_2_-KO) show inhibition of lung tumor initiation using a chemical carcinogenesis model [10]. In contrast, PGI_2_, which is also produced downstream from COX enzymes, has been shown to inhibit lung cancer initiation, as well as having anti-metastatic effects [11]. Increased expression of 5- and 12-lipoxygenase has also been associated with tumors, including lung cancer [12]. In contrast, expression of 15-lipoxygenase-2 appears to be lost in lung cancer, and may play an anti-tumorigenic role [13]. Lipoxygenase products have direct effects on tumor cells, but are also regulators of angiogenesis and can modify immune function [12]. Recently epoxyeicosatrienoic acids (EETs) produced through the cytochrome P450 pathway have been implicated as regulators of metastasis, acting at least in part through endothelial-specific effects at distant organs [14]. While combinations of COX and lipoxygenase inhibitors have been used as therapeutic agents and have shown beneficial effects in NSCLC [15], effects on metastasis have not been examined. In addition to studies focused on cancer cells, several reports have implicated eicosanoids, specifically PGE_2_, as regulators of the tumor microenvironment (TME) [16], [17]. PGE_2_ signaling through EP3/EP4 receptors leads to recruitment of cancer associated fibroblasts [18]. PGE_2_ has also been shown to be critical in promoting formation of T-regulatory lymphocytes [19]. Thus, cancer progression is likely to involve complex changes in multiple eicosanoids with potentially opposing biological effects. Both cancer cells and cells of the microenvironment are potent producers of eicosanoids, and tumor progression will depend on an integrated response to a spectrum of eicosanoids produced in a spatiotemporal fashion.

A limitation in lung cancer research is the lack of appropriate mouse models of metastatic lung cancer. We recently developed an immunocompetent orthotopic model in which murine lung cancer cells are directly implanted into the left lobe of syngeneic mice [20], [21]. These cells form a primary tumor which over a period of 3–5 weeks metastasizes to lymph nodes as well as liver and brain. Using this model, we have demonstrated that tumors growing in mice globally deficient in cPLA_2_ have a marked impairment in progression and metastasis [21]. Since the injected tumor cells expressed wild-type cPLA_2_, these data indicate that production of eicosanoids by cells of the tumor microenvironment is critical for lung cancer progression and metastasis. However, the critical eicosanoids mediating these effects have not been defined.

The advent of mass spectrometric analysis of lipids has allowed the measurement of multiple eicosanoid products from both cells and tissues [22]. This technique allows quantitative determination of a large number (>25) of distinct products. The goal of the present study was to apply mass spectrometric approach to systematically define changes in eicosanoids during lung cancer progression, and determine the role of distinct cell populations in creating this profile. Using immunocompetent orthotopic model in wild-type and cPLA_2_ knockout mice we are able to assess the contribution of the tumor microenvironment to production of individual eicosanoids. Our results demonstrate a complex eicosanoid pattern during tumor progression, and identify specific inflammatory cells in the TME that selectively contribute to eicosanoid production.

## Experimental Procedures

### Cells

Luciferase-expressing Lewis lung carcinoma cells (LLC-Luc) were obtained from ATCC and maintained in DMEM (Corning CellGro #19-017-CV) containing 10% fetal bovine serum, penicillin/streptomycin and G418 (500 ng/ml).

### Mice

cPLA_2_ knockout (cPLA_2_-KO) mice on a C57BL/6 background [21], [23] and wild type C57BL/6 mice were bred and maintained in the Center for Comparative Medicine at the University of Colorado Denver. Experiments were done in 12–16 week old mice, both males and females, with mice of different age and gender equally represented in all experimental groups. All procedures were performed under protocols approved by the Institutional Animal Care and Use Committee (IACUC) at the University of Colorado Denver.

### Orthotopic Mouse model

LLC-Luc cells (2×10^5^) were suspended in PBS containing 15% Matrigel (BD Biosciences) and injected into the parenchyma of the left lung lobe through the rib cage using a 30G needle. To directly visualize the lung a 4–5 mm incision was made in the skin under the left shoulder and subcutaneous fat was removed. After the procedure, the incision was closed using veterinary adhesive. Primary tumor size was measured using digital calipers.

### Extraction and mass spectrometry measurement of eicosanoids

Mice were sacrificed at 2 or 3 weeks after cancer cell injection and the whole left lung lobe containing the tumor was excised, weighed and flash-frozen in liquid nitrogen. Frozen tissues were homogenized in 1 ml of ice-cold sucrose buffer (250 mM sucrose, 50 mM HEPES, 1 mM EDTA, 1 mM EGTA) containing protease inhibitors using motor-driven homogenizer. Aliquots were taken for protein measurement. The remaining homogenate was immediately mixed with 2 volumes of ice-cold methanol. Protein was measured using Bradford reagent (Bio-Rad).

Eicosanoids were extracted and analyzed essentially as described previously, with some modifications [24]. After addition of stable isotope-labeled internal standards (2 ng each, except for the cys-LTs, of which 5 ng were added) to the methanol-containing homogenates, eicosanoids were extracted using solid phase cartridges (Strata-X 33 µm Polymeric Reversed Phase, Phenomenex), eluted with methanol, dried and resuspended in 60 µL of 2 volumes of solvent A (8.3 mM ammonium acetate, pH 5.7) and 1 volume of solvent B (acetonitrile/methanol 65∶35 v/v). A 25 µL-aliquot was subsequently analyzed by LC-MS/MS using an Ascentis C18 HPLC column (150×2 mm, 5 µm; Supelco) interfaced with the electrospray source of a triple quadrupole mass spectrometer (Sciex API 3000; PE-Sciex, Thornhill, Ontario, Canada). Samples were eluted at a flow rate of 200 µL/min with a linear gradient from 25% to 100% of solvent B, which was increased from 25% to 85% in 24 min, to 100% in 26 min, and held at 100% for a further 12 min. Mass spectrometric analysis was performed in the negative ion mode using multiple reaction monitoring of the following specific *m/z* transitions:

369.300→169.100TXB_2_

369.300→163.1006-keto-PGF_1α_

353.300→193.100PGF_2α_

351.300→271.200PGE_2_

351.300→233.200PGD_2_

333.300→189.100PGA_2_ and PGJ_2_

315.300→203.20015deoxy-PGJ_2_

351.300→217.200LXA_4_

351.300→221.200LXB_4_

335.300→195.100LTB_4_

335.200→115.1005,6-diHETEs

365.300→195.10020-COOH-LTB_4_

351.300→195.10020-OH-LTB_4_

624.500→272.200LTC_4_

495.400→177.100LTD_4_

438.300→333.300LTE_4_

319.300→115.1005HETE

335.300→203.2005HpETE

317.300→203.2005oxoETE

319.300→179.10012HETE

319.300→219.20015HETE

343.300→245.20017OH-DHAa

343.300→273.20017OH-DHAb

327.300→283.200DHA

303.300→205.200AA

319.200→191.1005,6-EET

319.200→155.1008,9-EET

319.200→208.10011,12-EET

319.200→175.10014,15-EET

373.300→173.100[^2^H_4_]-TXB_2_

357.300→197.100[^2^H_4_]-PGF_2α_

373.300→167.100[^2^H_4_]-6-keto-PGF_1α_

355.300→275.200[^2^H_4_]-PGE_2_

339.300→197.100[^2^H_4_]-LTB_4_

629.500→272.200[^2^H_5_]-LTC_4_

500.300→177.100[^2^H_5_]-LTD_4_

443.300→338.300[^2^H_5_]-LTE_4_

327.300→116.100[^2^H_8_]-5HETE

311.300→267.200[^2^H_8_]-AA

Quantitation was performed using standard isotope dilution as described previously [25]

### Preparation of single cell suspension and sorting by flow cytometry

Mice were sacrificed at 2.5 weeks after cancer cell injection, the circulation was perfused with PBS/heparin (80 U/ml, Sigma), and left lung lobes containing tumors were excised. Tissues from 4 mice were pooled, mechanically dissociated, incubated at 37°C for 40 minutes with Collagenase A (1 mg/ml, Roche) and DNAse I (40 µg/ml, Sigma), and processed using GentleMACS dissociator C tubes (Miltenyi Biotec). Resulting single-cell suspensions were filtered through 70 µm cell strainers (BD), subject to red blood cells lysis using hypotonic buffer, and filtered second time through 40 µm cell strainers. Prior to staining, FcγR was blocked with anti-CD16/CD32 (BD Biosciences) for 20 minutes. Cells were stained for 1 h at 4°C with the following antibodies: CD11b-FITC (Clone M1/70, BD Biosciences), SiglecF-PE (Clone E50-2440, BD Biosciences), Ly6G-PE-Cy7 (Clone 1A8, BD Biosciences), F4/80-APC (Clone CI:A3-1, AbDSerotec), CD11c-APC-Cy7 (Clone HL3, BD Biosciences). Cells were sorted at the University of Colorado Cancer Center Flow Cytometry Core using XDP-100 (Beckman Coulter). The sorting strategy involved excluding debris and cell doublets by light scatter, and dead cells by DAPI (1 µg/ml). Data was analyzed using Kaluza Software (Beckman Coulter).

### Treatment of flow cytometry-sorted cells for measurement of eicosanoid production

Immediately after sorting, 1.5 – 2.0×10^5^ of cells of each type were pelleted, resuspended in PBS without serum and treated with calcium ionophore (A23187, 0.5 µM) for 12 minutes. Reactions were terminated by the addition of ice-cold methanol.

### Quantitative real-time-PCR

Total RNA was isolated from flow cytometry-sorted cells using the QIAshredder and RNeasy Micro kit (Qiagen). Extracted RNA was converted into complementary DNA with the iScript cDNA synthesis kit (BioRad). Real time PCR was performed using the MyIQ Real Time PCR Detection System (BioRad) with the following primers : cPLA_2_: Fwd 5′-GTGGTGGCCATTTTGGGTTC-3′, Rev 5′-TCGGGGTGAGAGTACAAGGT-3′, COX-2: Fwd 5′-TGAGCAACTATTCCAAACCAGC-3′, Rev 5′-CACGTAGTCTTCGATCACTATC-3′, COX-1: Fwd 5′- ATGGATACTGGCTCTGGGAA -3′, Rev 5′- CTGAGTTGTAGGTCGGAGGG -3′, 5-LO: Fwd 5′- ACTACATCTACCTCAGCCTCATT-3′, Rev 5′- GGTGACATCGTAGGAGTCCAC-3′, LTC4S: Fwd 5′- GTCTTCCGAGCCCAGGTAAA-3′, Rev 5′- CGCGCGTATCCCTGGAAATA-3′, LTA4H: Fwd 5′- ATCTCTCTTCCCATCGCCCT-3′, Rev 5′-CTTTCGGGACAGACACCTCT-3′, TXAS1: Fwd: 5′- GCTTCCACCTTCTGTATCCC-3′, Rev:5′- TCTCGGTTCTTATTGGGCAG-3′, MAGL: Fwd: 5′- CGAACTCCACAGAATGTTCCCTA -3′, Rev: 5′- ACAAAGATGAGGGCCTTGGGT - 3′, β-actin: Fwd: 5′- GGCTGTATTCCCCTCCATCG-3′, Rev 5′-CCAGTTGGTAACAATGCCATG- 3′, GAPDH: Fwd 5′- CGTGGAGTCTACTGGTGTCTTC - 3′, Rev 5′- CGGAGATGATGACCCTTTTGGC -3′ Ubiquitin C: Fwd: 5′- CCACACAAAGCCCCTCAATC-3′, Rev: 5′- AAAGATCTGCATCGTCTCTCTCAC-3′.

### Statistical & Computational Analyses

We used Friedman's Chi-Square test analysis to evaluate the difference between tumor-bearing lungs from WT and cPLA_2_-KO mice harvested at 3 weeks. Rank scores, instead of raw eicosanoid level measurements, were used in the test. We applied False Discovery Rate (FDR) control to the p-values obtained from Chi-Square test, in order to correct for multiple comparisons. The FDR values are considered "adjusted" p-values. We concluded that an association is significant when the FDR value is less than 0.05.

## Results

### Multiple eicosanoids are increased in tumor bearing mice

Using an orthotopic immunocompetent mouse model, in which tumor cells are injected into lungs of mice, we previously showed that loss of cPLA_2_ in the tumor microenvironment inhibited lung cancer metastasis[21]. These effects are presumably due to altered production of specific eicosanoids critical for tumor progression and metastasis. To define the spectrum of eicosanoids produced during lung tumor progression in orthotopic lung cancer model we used liquid chromatography coupled with tandem mass spectrometry (LC/MS/MS).

Lewis Lung Carcinoma cells (LLC-Luc: 2×10^5^), derived from a lung adenocarcinoma in a C57BL/6 mouse, were injected into the left lung lobes of syngeneic WT mice. Whole left lung lobes containing tumors were harvested at 2 and 3 weeks after injection, along with left lobes from control mice injected with PBS/Matrigel. Eicosanoids extracted from the harvested lungs were analyzed by LC/MS/MS and normalized to the protein content of the sample. We monitored multiple products of the cyclooxygenase (COX), lipoxygenases (LO), and cytochrome P450 pathways. Additionally, we measured free arachidonic acid (AA) and docosahexaenoic acid (DHA).

**Figure 1:**
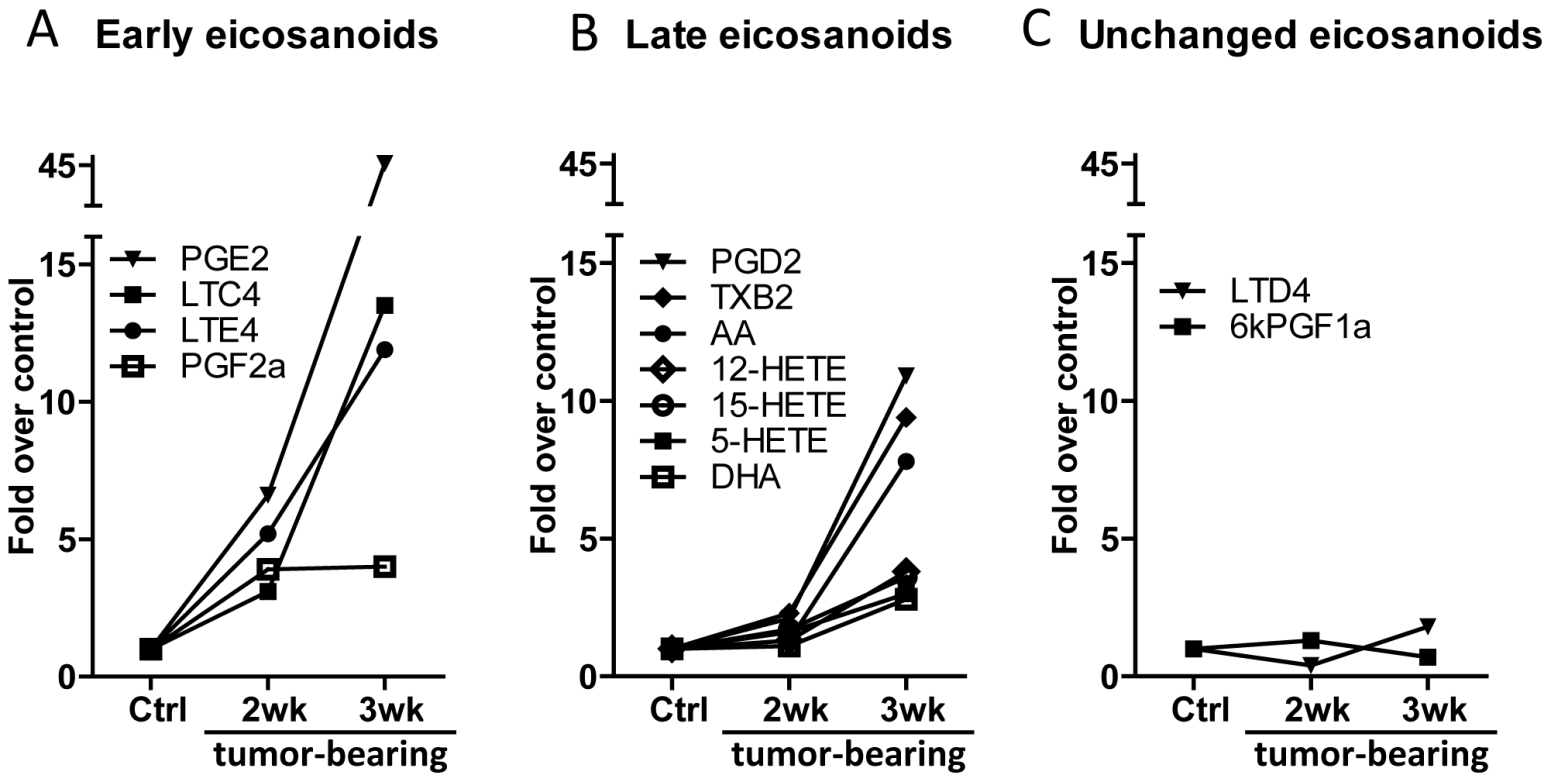
Multiple eicosanoids are increased in tumor bearing mice. LLC-Luc cells or matrigel (control) were injected into left lung lobes of WT C57Bl/6 mice and tumor-bearing and control lungs were harvested 2 or 3 weeks after injection. Eicosanoid levels were analyzed by LC/MS/MS and normalized to the protein content of the sample. Levels on graphs are expressed as fold over control. A. Early eicosanoids – induced >3 fold at 2 weeks, and further increasing at 3 weeks. B. Late eicosanoids – not upregulated (<2-fold) at 2 weeks, but increased at 3 weeks. C. Unchanged eicosanoids.

In WT mice levels of eicosanoids were generally increased with cancer progression, however with various degrees of induction and different temporal patterns (Table 1 and Fig.1). We differentiated a group of eicosanoids that were up-regulated 3–7 fold at the earlier 2-week time point (early eicosanoids): PGE_2_, PGF_2α_, LTC_4_ and LTE_4_ (Fig. 1A). Levels of this subset of eicosanoids were further increased at 3 weeks. Compared to Matrigel-injected mice, PGE_2_ was upregulated 45-fold, which was the highest increase of all eicosanoids. LTC_4_ and LTE_4_ were also highly increased, 12 and 13 fold, respectively. The levels of PGF_2α_ did not further increase at 3 weeks. A second group included eicosanoids that were not increased (< 2-fold) at 2 weeks, but increased significantly at 3 weeks (late eicosanoids, Fig. 1B). Some of the late eicosanoids: PGD_2_, TXB_2_ (the stable metabolite of TXA_2_) and AA became highly upregulated (7–10 fold compared to Matrigel injected), while others (5-, 12-, 15-HETE, and DHA) were upregulated moderately (3–4 fold). LTB_4_, which was undetectable at the 2 week time point, was detected in 50% of the animals at 3 weeks. Two of the eicosanoids: LTD_4_ and 6-keto-PGF_1α_ (the stable metabolite of PGI_2_), were unchanged or only marginally upregulated at either 2 or 3 weeks (Fig. 1C). Neither lipoxins nor the cytochrome P450 products (5,6-EET, 8,9-EET, 11,12-EET, 14,15-EET), were detected at any time points.

**Table 1:** Increase in eicosanoid levels during lung tumor progression in orthotopic lung cancer model in mice.

analyte	Fold increase in 3 wk tumors over control	FDR (adj. p-value)
LTB4	ND	0.218
PGE2	45.2	0.009*
LTC4	13.5	0.212
LTE4	11.9	0.009*
PGD2	10.9	0.010*
TXB2	9.4	0.010*
AA	7.8	0.009*
PGF2a	4.0	0.065
12HETE	3.8	0.010*
15HETE	3.6	0.009*
5HETE	3.0	0.263
DHA	2.8	0.009*
5-oxo-ete	2.4	0.182
LTD4	1.8	0.263
6kPGF1a	0.7	0.699

Wild-type C57B/L mice were injected in left lung lobe with LLC-Luc cells. Whole left lung lobes containing tumors were harvested at 2 and at 3 weeks after injection, along with left lobes from control mice injected with PBS/Matrigel. Eicosanoids extracted from the harvested lungs were analyzed by LC/MS/MS and normalized to the protein content of the sample. Numbers in the table indicate fold increase in 3-week tumor bearing lung vs. matrigel-injected lung. ND – fold increase was not determined for LTB4, since in control mice this analyte was not detectable. * FDR<0.05

### Deletion of cPLA_2_ in the TME differentially affects eicosanoids

Since cPLA_2_ is the rate-limiting enzyme in eicosanoid biosynthesis, we measured eicosanoid production in lung tumors grown in cPLA_2_ –knockout mice. To this end, at the same time that the WT mice were injected, we also injected vehicle (Matrigel) or LLC-Luc cells into mice globally deficient in cPLA_2_. With this experimental strategy, cPLA_2_ is deleted in all cells of tumor microenvironment, while wild-type expression is maintained in the cancer cells. Animals were sacrificed at 2 and 3 weeks after injection and eicosanoid profiles in tumor-bearing or control left lung lobes were determined by LC/MS/MS.

**Figure 2:**
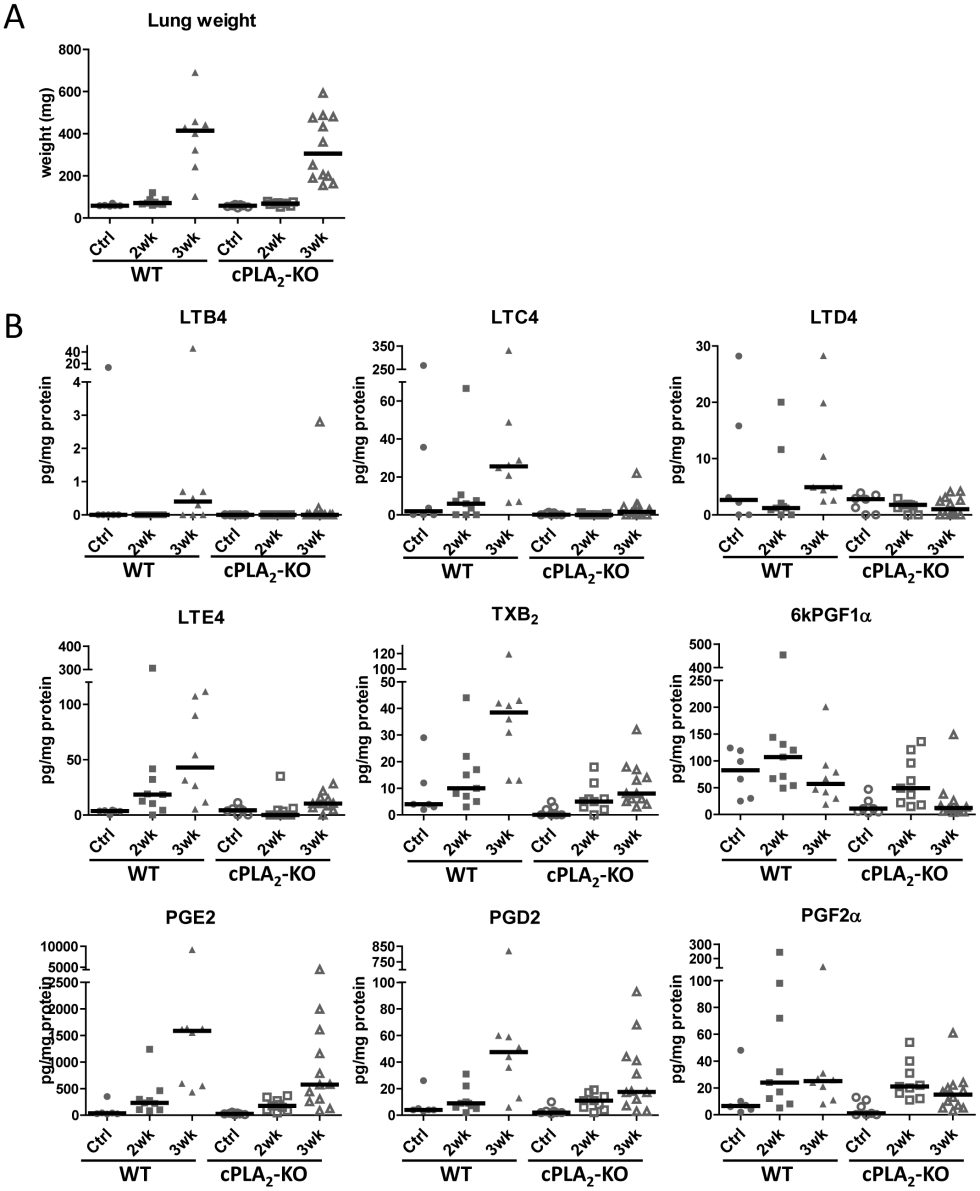
Deletion of cPLA_2_ in the TME differentially affects eicosanoid production. LLC-Luc cells or matrigel (control) were injected into left lung lobes of WT or cPLA_2_-KO mice. Injections were done on 3 separate days with control vs. tumor and WT vs. cPLA_2_-KO groups equally represented on each day. Left lobes harboring tumors and control lungs were harvested 2 or 3 weeks after injection, eicosanoids were analyzed by LC/MS/MS and normalized to protein content of the sample. Each point represents a single mouse. Bars represent medians. A. Weights of tumor-bearing and control lungs B. Select eicosanoids

As shown in Fig. 2A, growth of primary tumors was not different in wild-type vs. cPLA_2_-KO mice, consistent with our previous observation [21]. Analysis of eicosanoid profiles (Table 2 and Fig. 2B) revealed that basal levels of eicosanoids in cPLA_2_-KO mice were in general slightly lower, but not statistically different from WT control mice. With lung tumor progression, eicosanoid production increased in both WT and cPLA_2_-KO mice; however, that increase was significantly blunted in tumor-bearing cPLA_2_-KO mice. The level of inhibition in cPLA_2_-KO mice varied widely among the various analytes, ranging from about 25% to over 90% inhibition. This result indicates that one can make a distinction between eicosanoids that are produced primarily by the microenvironment (completely inhibited in the setting of cPLA_2_ knockout), and eicosanoids contributed both by microenvironment and the cancer cells (reduced partially). Detailed analysis of the eicosanoid profiles revealed that AA and DHA showed the slightest reduction associated with cPLA_2_ deficiency, about 25% at 3 weeks (Table 2). 5-, 12- and 15-HETE were on average reduced by 50% in cPLA_2_-KO mice compared to WT mice (Table 2). Products of COX pathway showed various degrees of inhibition in the setting of cPLA_2_ deficiency. While TXB_2_ and 6kPGF_1α_ were reduced by 80% at 3 weeks, the other prostaglandins (PGE_2_, PGD_2_ and PGF_2a_) were on average 50% lower than in WT mice (Table 2 and Fig. 2B). The largest inhibition associated with cPLA_2_-deficiency in the microenvironment was detected in the leukotriene group (Table 2 and Fig. 2B). The increase in early leukotrienes, LTC_4_ and LTE_4_, was delayed until 3 weeks in cPLA_2_-KO mice compared to WT mice, and their levels were reduced by 90% for LTC_4_ and 75% for LTE_4_. Leukotriene LTB_4,_ which was induced in 3wk WT tumor-bearing lungs, was not detected in cPLA_2_-KO mice at any time point. The levels of LTD_4_, while not increased with tumor progression, were lower by 80% in 3-week cPLA_2_-KO tumors.

**Table 2:** Eicosanoid levels in tumor-bearing lungs of WT and cPLA2-KO mice.

	WT	WT	WT	cPLA2-KO	cPLA2-KO	cPLA2-KO		
analyte	No tumor	Tumor 2wks	Tumor 3wks	No tumor	Tumor 2wks	Tumor 3 wks	% reduction KO vs WT in 3 wk tumors	FDR (adj. p-value)
DHA	1.5	1.6	4.1	1.1	1.2	3.1	24	0.185
	(1.3–1.9)	(1.3–2.1)	(3.5–6.0)	(1.0–1.4)	(1.1–1.5)	(2.4–4.7)		
AA	8	10	63	6	8	47	25	0.172
	(8–10)	(9–11)	(50–66)	(5–7)	(7–9)	(38–58)		
PGF2a	6	24	25	1	21	15	41	0.094
	(4–10)	(12–72)	(16–29)	(0–11)	(16–31)	(6–21)		
12HETE	102	136	384	45	111	210	45	0.072
	(56–263)	(112–279)	(333–540)	(37–117)	(76–191)	(138–354)		
15HETE	40	67	143	18	52	60	58	0.139
	(24–60)	(56–123)	(108–155)	(11–49)	(32–79)	(35–156)		
5HETE	6	9	18	4	3	7	61	0.355
	(4–23)	(9–12)	(9–60)	(2–5)	(3–7)	(4–30)		
PGD2	4	9	47	2	11	18	63	0.185
	(4–5)	(6–10)	(25–59)	(2–3)	(6–14)	(10–42)		
PGE2	35	232	1587	25	176	575	64	0.182
	(26–45)	(104–300)	(574–1634)	(11–41)	(99–275)	(279–1382)		
LTE4	4	19	43	4	0	10	76	0.031*
	(3–4)	(10–32)	(19–99)	(0–6)	(0–4)	(7–15)		
TXB2	4	10	38	0	5	8	78	0.015*
	(3–12)	(7–17)	(22–43)	(0–3)	(3–6)	(5–15)		
LTD4	3	1	5	3	2	1	79	0.012*
	(0–16)	(1–2)	(4–15)	(0–4)	(1–2)	(0–3)		
6kPGF1a	82	107	57	11	49	12	79	0.015*
	(30–119)	(67–131)	(36–85)	(5–25)	(22–96)	(6–21)		
LTC4	2	6	26	0	0	2	94	0.007*
	(0–36)	(0–8)	(14–39)	(0–1)	(0–0)	(0–5)		
LTB4	0	0	0.36	0	0	0	100	0.075
	(0–0)	(0–0)	(0–0.68)	(0–0)	(0–0)	(0–0)		

LLC-Luc cells or matrigel (control) were injected into left lung lobes of wild-type or cPLA_2_-KO mice. Left lobes with tumor were harvested 2 or 3 weeks after injection, eicosanoids were analyzed by LC/MS/MS and normalized to protein content of the sample. Top numbers indicate median levels of each eicosanoid. Numbers in parentheses indicate the interquartile ranges. Eicosanoid levels are expressed as pg/mg protein, except for DHA and AA, which are measured in ng/mg protein. *FDR value <0.05

### Distinct populations of inflammatory cells in the TME have different eicosanoid profiles

**Figure 3:**
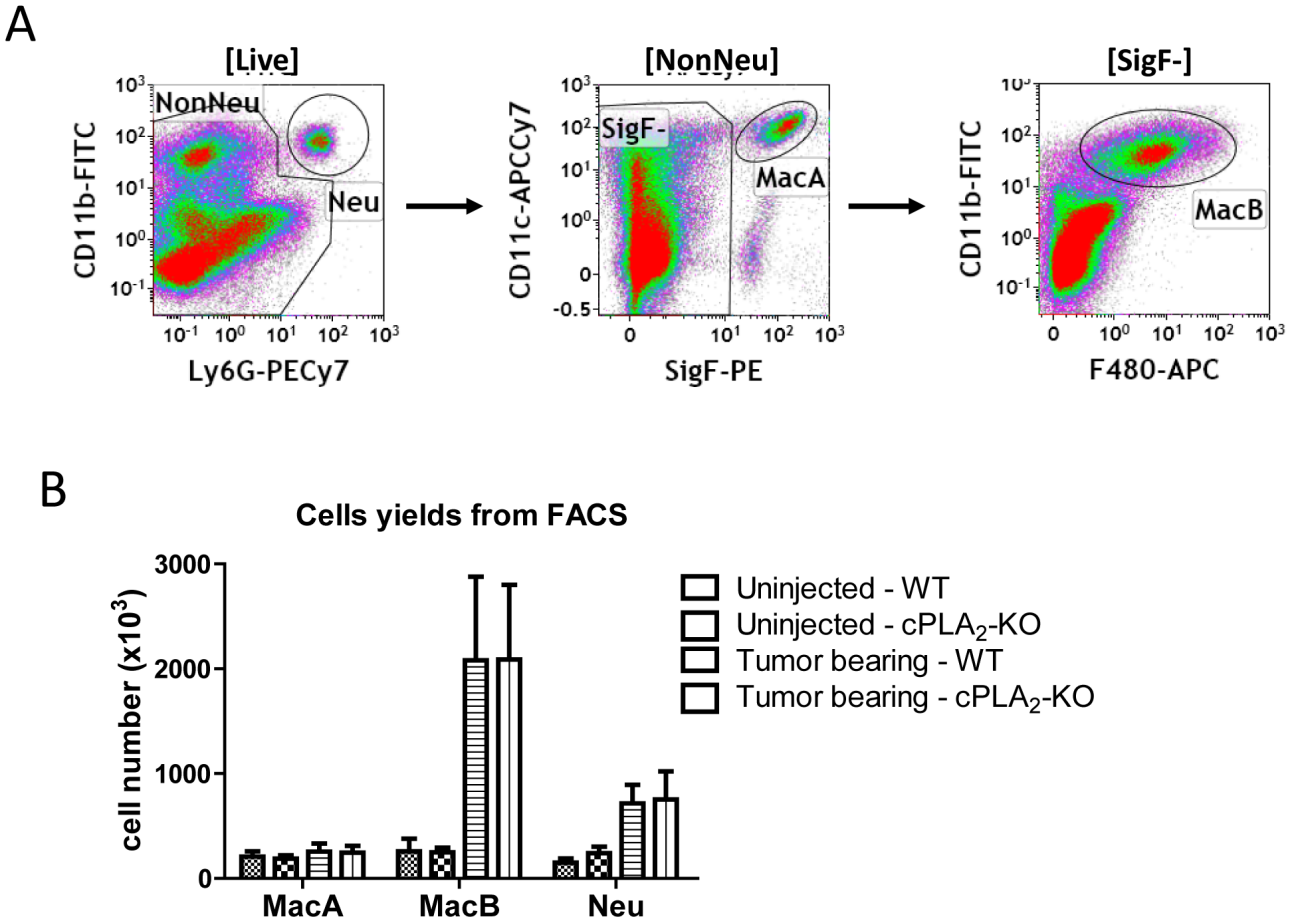
Recovery of inflammatory cells from TME by flow cytometry. LLC-Luc cells were injected into left lung lobes of WT or cPLA_2_-KO mice and tumor-bearing lungs were harvested 2.5 weeks later. Tissues from 4 mice were pooled, and inflammatory cells were recovered by flow cytometry. A. Sequential flow cytometry gating strategy used to recover inflammatory cells: Neu: neutrophils (CD11b+/Ly6G+), MacA macrophages (SigF+/CD11c+/Ly6G-) and MacB macrophages (F480+/CD11b+/Ly6G-/SigF-). B. Numbers of cells recovered by flow cytometry from uninjected or tumor-bearing left lung lobes of WT and cPLA_2_-KO mice. Data show average from 3 separate experiments, with each sorting performed on a pool of 4 mice. Error bars show S.E.M.

The TME is composed of multiple cell types, including inflammatory cells, fibroblasts and adaptive immune cells. In order to begin to define which of these cell populations are producing leukotrienes in the setting of tumor progression, we used flow cytometry to isolate inflammatory cells from lungs of tumor-bearing mice. Using markers previously employed to characterize inflammatory cells in the lung, we defined neutrophils (Neu, CD11b+/Ly6G+), as well as two populations of macrophages, designated as MacA (SigF+/CD11c+/F480+/CD11b low) and MacB (F480+/CD11b+/Ly6G-/SigF-), (Fig. 3A). Based on previous studies [26], [27], MacA represent resident alveolar macrophages [27], whereas MacB is a heterogeneous population mainly composed of infiltrating monocytes and macrophages, and also contains interstitial macrophages, and CD11b+ dendritic cells [27], [28]. In the setting of tumors, the number of MacB cells was markedly increased, whereas the MacA population was not significantly altered compared to control mice not injected with cancer cells (Fig. 3B).

**Figure 4:**
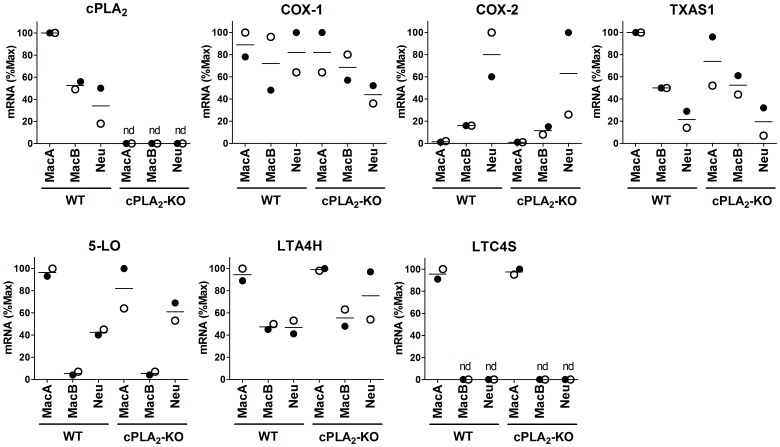
Expression of enzymes in the eicosanoid pathway in inflammatory cells recovered from tumor-bearing lungs. Relative mRNA expression in cells recovered from tumor-bearing lungs by flow cytometry. Expression was assessed by qRT-PCR, normalized to the geometric average of β-actin, GAPDH, Ubiquitin C, and 18s, and expressed as % Maximum among the analyzed cell groups. Experiments were performed on 2 separately injected and analyzed pools of mice (4 mice per WT or cPLA2-KO group per pool). Filled circles – pool #1, open circles – pool #2, bar – mean of the pools.

**Figure 5:**
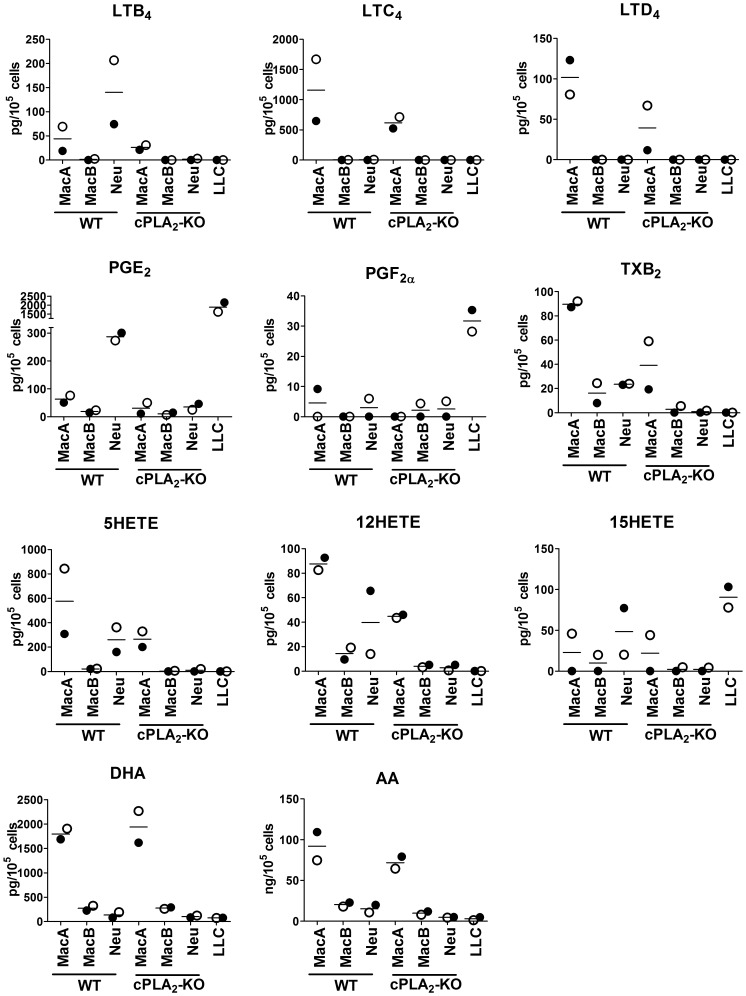
Eicosanoid production by inflammatory cells recovered by flow cytometry. Eicosanoid production in cells recovered from tumor-bearing lungs by flow cytometry. Recovered cells were stimulated with ionophore A23187 (0.5 µM) for 12 minutes. Extracted eicosanoids were analyzed by LC/MS/MS. Experiments were performed on 2 separately injected and analyzed pools of mice (4 mice per WT or cPLA2-KO group per pool). Filled circles – pool #1, open circles – pool #2, bar – mean of the pools.

To determine the potential for eicosanoid production by these three groups of inflammatory cells, we recovered them by flow cytometry from lungs of tumor-bearing mice, and examined mRNA expression of enzymes in the eicosanoid pathway by qRT-PCR. The analysis of mRNA revealed significant differences in the expression of eicosanoid pathway enzymes between different inflammatory cells (Fig. 4). All three populations expressed cPLA_2_, although levels were higher in MacA cells than in MacB or neutrophils. COX-1 was also expressed in all 3 cells types, and we did not detect significant differences in its levels, suggesting that all of these populations can produce prostaglandins. However, COX-2 was selectively expressed in neutrophils, with low levels in MacB and almost undetectable in MacA. Since inflammatory cytokines induce prostaglandins through increased COX-2 expression, we would predict that prostaglandin production would be mediated largely through neutrophils and MacB. Thromboxane synthase TXAS1 was also detected in all three cell types. 5-LO was highest in MacA cells, Neutrophils expressed 50–70% lower levels, and expression in MacB was 90% lower than in MacA. This would indicate that leukotriene production in the setting of tumor progression is largely derived from MacA or neutrophils, with the recruited MacB population contributing very little. LTA4H was expressed in all 3 cell types, while the expression of LTC4S was restricted only to MacA cells. All the analyzed enzymes were expressed at the same levels in WT and cPLA_2_-KO mice, with the exception of cPLA_2_, which, as expected, was undetectable in cells derived from cPLA_2_-KO. Since free AA may be released by enzymes other than cPLA_2_, such as monoacyl glycerol lipase (MAGL)[29] we measured MAGL levels in isolated cells. MacA cells demonstrated high levels of MAGL compared to either MacB cells or neutrophils (data not shown). This result leads to prediction that cPLA_2_ deficiency will have less impact on eicosanoid production by MacA cells than by MacB or neutrophils.To confirm the potential for eicosanoid production by MacA, MacB and neutrophil populations, cells were recovered from tumor-bearing mice by flow cytometry and stimulated with calcium ionophore A23187. Eicosanoid production was assessed by LC/MS/MS (Fig.5). Along with the sorted cells, we examined eicosanoid production by cultured LLC-Luc cancer cells. The largest repertoire of eicosanoids was produced by MacA cells. In agreement with their high expression of 5-LO and exclusive expression of LTC4S, MacA were the only ones capable of producing cysteinyl leukotrienes, LTC_4_ and LTD_4_. MacA also produced LTB_4_, albeit less than neutrophils, 5-HETE, and 5oxoETE. Despite their low expression of COX-2, but consistent with expression of COX-1, MacA cells were able to produce PGE_2_ and TXB_2_. Interestingly, eicosanoid production by MacA cells from cPLA_2_-KO mice was reduced compared to WT, but only by about 50%. These data suggest that additional pathways distinct from cPLA_2_, such as MAGL act to provide AA in these cells. MacB cells produced lower levels of eicosanoids. As they lack 5-LO, MacB were not capable of producing leukotrienes. Despite expression of enzymes in the cyclooxygenase pathway, MacB produced only low levels of PGE_2_ and TXB_2_, which were reduced by 80% in the setting of cPLA_2_-KO. Neutrophils, which express 5-LO, but not LTC4S, produced LTB_4_ at levels 3-fold higher than MacA, but no cysteinyl leukotrienes. Consistent with their high expression of COX-2, neutrophils produced PGE_2_, at levels 4-fold higher than MacA. They also produced 5-HETE and TXB_2_, at levels lower than MacA cells. In the setting of cPLA_2_ deficiency, neutrophil production of eicosanoids was reduced over 90% in comparison to WT mice. Among other metabolites, 12-HETE, PGF_2α_, 15-HETE, AA, and DHA were produced by all 3 cell types. In contrast to myeloid cells recovered from the TME, LLC-Luc cells produced a limited spectrum of eicosanoids. Their major product was PGE_2_, detected at levels about 10 times higher than in myeloid cells. We also detected PGF_2α_, 15-HETE and relatively low levels of DHA and AA. No leukotrienes were detected, consistent with our in vivo data indicating that leukotrienes are selectively produced by the TME.

## Discussion

Eicosanoids represent a large family of bioactive signaling molecules with diverse and potentially opposing biological effects. While much work has focused on specific eicosanoids and their role in cancer, there has not been a systematic attempt to define the spectrum of these molecules in the setting of cancer. A large body of literature has defined both pro- and anti-tumorigenic roles for products of both the cyclooxygenase and lipoxygenase pathways [30], [31]. Our studies used an immunocompetent orthotopic model of lung cancer progression, combined with a lipidomic mass spectrometry approach to demonstrate that lung cancer progression is associated with increases in multiple eicosanoid products derived from both the cyclooxygenase and lipoxygenase pathways. Individual eicosanoids showed different time dependencies, with a subset being increased at the earliest time point analyzed, whereas a second subset appeared to have a more delayed course of induction. Several products (e.g. 6k-PGF_1α_) were not changed, and products of the cytochrome P450 pathway or lipoxins were not detected in our model. While lung cancer initiation is mediated through regulation of oncogenic and tumor suppressor pathways, progression and metastasis involve complex interactions between the tumor cells and the TME [32]. Increased eicosanoid production during tumor progression may be a result of increasing numbers of inflammatory cells in the microenvironment. In addition, studies in many types of cancer have demonstrated that inflammatory cells undergo phenotypic modulation. Circulating monocytes/macrophages recruited to a tumor have been reported to either exhibit a tumoricidal phenotype, or upon contact with cancer cells, switch to a tumor-promoting phenotype [33], [34]. The eicosanoid profiles of inflammatory cells may change during this phenotypic switch, leading to different induction of specific eicosanoids at later time points.

Since cPLA_2_ is critical for eicosanoid production in most cell types, comparison of tumors grown in WT vs. cPLA_2_-KO mice allowed us to assess the contribution of the TME to eicosanoid production. In cPLA_2_-KO mice, we observed almost complete inhibition of leukotriene production, indicating that these products are largely produced by TME cells and their production is dependent on cPLA_2_. In contrast, levels of PGE_2_ and other COX products were only moderately decreased (<50%) compared to tumors grown in WT mice, and were still increased compared to non-tumor bearing cPLA_2_-KO mice. This result can be attributed to continued eicosanoid production by LLC cells, which produce large amounts of PGE_2_ in vitro. Thus our data demonstrate that PGE_2_ is produced by both tumor cells and stromal cells. From our analysis we would conclude that production of most cyclooxygenase products involves both the tumor cells and the TME, but that leukotrienes are largely produced by the TME.

Using novel flow cytometry approaches developed for examining innate immune cells in the lung [26]-[28] we have been able to quantify changes in several distinct populations during cancer progression. Both the number of interstitial and infiltrating macrophages (MacB) and neutrophils were increased during tumor progression, whereas the number of resident alveolar macrophages (MacA) was not altered. While the contribution of a particular cell type to eicosanoid production will depend on the relative cell numbers in the setting of tumor, the in vivo stimuli, as well as on localization and timing of eicosanoid secretion, our data indicate that subtypes of inflammatory cells have different capacities for specific eicosanoids.

MacA cells, which represent resident alveolar macrophages, produced high levels and a large array of eicosanoids. Indeed, they were the only group capable of producing cysteinyl leukotrienes. Interestingly, in the setting of cPLA_2_ knockout, eicosanoid production by MacA was significantly reduced, but not completely abrogated, which correlates with the reduction detected in the whole tumor-bearing lung. This result confirms critical role of cPLA_2_ in eicosanoid production, but also indicates that MacA cells may use other sources of free AA, possibly monoacylglycerol lipase for eicosanoid generation [35]. Since the numbers of resident alveolar macrophages do not significantly increase during tumor progression (Fig. 3B), increased leukotriene and thromboxane levels in the whole tumor-bearing lung could be due to the activation of these cells.

Surprisingly, in MacB cells, which include mainly infiltrating monocytes/macrophages, the eicosanoid production per-cell was relatively low, despite clear expression of several enzymes in the eicosanoid pathway. Nonetheless, these cells may still significantly contribute to overall eicosanoid levels in the TME, if one considers high MacB cell infiltration during tumor progression (Fig. 3B). The low detected eicosanoid levels in MacB cells in vitro may also be due limitations of the in vitro assay. While increases in intracellular Ca^2+^ as a result of exposure to ionophore will increase cPLA_2_ activity, in many cells stimulation of additional pathways by growth factors or cytokines is required for maximal eicosanoid production [36], [37]. In fact, stimulation of MacB cells with a combination of ionophore and phorbol ester (PMA) led to increased production of several eicosanoids, such as PGE_2_ and HETEs compared to ionophore alone (not shown). Thus in vivo, MacB may be exposed to a milieu which activates these pathways. Alternatively, MacB cells may be specialized to perform functions other than eicosanoid production, such as cytokine secretion. In fact, these cells express high levels of Arginase-I and IL-1β, which in turn are very low in MacA or neutrophils (not shown). The analysis of tumor-associated neutrophils revealed that these cells had the highest per-cell production of LTB_4_ and PGE_2_, and the production of these metabolites by neutrophils was wholly dependent on cPLA_2_. Our analysis focused on myeloid cells, since these cells were shown to produce high levels of eicosanoids in other settings, but full understanding of eicosanoid production in TME would require analysis of other cell types. Interestingly, we have not detected measureable levels of cytochrome P450 products in our tumors. Production of these eicosanoids by endothelial cells has been implicated in regulating distant organ metastases [14], suggesting that they may be produced in a localized fashion.

In conclusion, our studies, using an immunocompetent orthotopic model of lung cancer and combining flow cytometry and mass spectrometry technologies, define a complex profile of eicosanoid production during tumor progression, in which both cancer cells and specific cells of the microenvironment produce distinct eicosanoid products. Consequently, our studies implicate that anti-eicosanoid therapies will have differential effect on the cancer cells and on the cells of tumor microenvironment and provide a rationale to develop strategies targeting these populations to inhibit specific classes of eicosanoids.

## References

[1] Nakanishi M, Rosenberg DW (2006) Roles of cPLA2alpha and arachidonic acid in cancer. Biochim Biophys Acta 1761: 1335–1343. 17052951 10.1016/j.bbalip.2006.09.005 PMC1761949

[2] Schneider C, Pozzi A (2011) Cyclooxygenases and lipoxygenases in cancer. Cancer Metastasis Rev 30: 277–294. 22002716 10.1007/s10555-011-9310-3 PMC3798028

[3] Buczynski MW, Dumlao DS, Dennis EA (2009) Thematic Review Series: Proteomics. An integrated omics analysis of eicosanoid biology. J Lipid Res 50: 1015–1038. 19244215 10.1194/jlr.R900004-JLR200 PMC2681385

[4] Narumiya S, Sugimoto Y, Ushikubi F (1999) Prostanoid receptors: structures, properties, and functions. Physiol Rev 79: 1193–1226. 10508233 10.1152/physrev.1999.79.4.1193

[5] Jemal A, Siegel R, Ward E, Hao Y, Xu J, et al (2008) Cancer statistics, 2008. CA Cancer J Clin 58: 71–96. 18287387 10.3322/CA.2007.0010

[6] Hida T, Yatabe Y, Achiwa H, Muramatsu H, Kozaki K, et al (1998) Increased expression of cyclooxygenase 2 occurs frequently in human lung cancers, specifically in adenocarcinomas. Cancer Res 58: 3761–3764. 9731479

[7] Wolff H, Saukkonen K, Anttila S, Karjalainen A, Vainio H, et al (1998) Expression of cyclooxygenase-2 in human lung carcinoma. Cancer Res 58: 4997–5001. 9823297

[8] Heasley LE, Thaler S, Nicks M, Price B, Skorecki K, et al (1997) Induction of cytosolic phospholipase A2 by oncogenic Ras in human non-small cell lung cancer. J Biol Chem 272: 14501–14504. 9169405 10.1074/jbc.272.23.14501

[9] Moody TW, Leyton J, Zakowicz H, Hida T, Kang Y, et al (2001) Indomethacin reduces lung adenoma number in A/J mice. Anticancer Res 21: 1749–1755. 11497255

[10] Meyer AM, Dwyer-Nield LD, Hurteau GJ, Keith RL, O'Leary E, et al (2004) Decreased lung tumorigenesis in mice genetically deficient in cytosolic phospholipase A2. Carcinogenesis 25: 1517–1524. 15033900 10.1093/carcin/bgh150

[11] Keith RL, Miller YE, Hoshikawa Y, Moore MD, Gesell TL, et al (2002) Manipulation of Pulmonary Prostacyclin Synthase Expression Prevents Murine Lung Cancer. Cancer Res 62: 734–740. 11830527

[12] Pidgeon GP, Lysaght J, Krishnamoorthy S, Reynolds JV, O'Byrne K, et al (2007) Lipoxygenase metabolism: roles in tumor progression and survival. Cancer Metastasis Rev 26: 503–524. 17943411 10.1007/s10555-007-9098-3

[13] Gonzalez AL, Roberts RL, Massion PP, Olson SJ, Shyr Y, et al (2004) 15-Lipoxygenase-2 expression in benign and neoplastic lung: an immunohistochemical study and correlation with tumor grade and proliferation. Hum Pathol 35: 840–849. 15257547 10.1016/j.humpath.2004.04.001

[14] Panigrahy D, Edin ML, Lee CR, Huang S, Bielenberg DR, et al (2011) Epoxyeicosanoids stimulate multiorgan metastasis and tumor dormancy escape in mice. J Clin Invest 122: 178–191. 22182838 10.1172/JCI58128 PMC3248288

[15] Soriano AF, Helfrich B, Chan DC, Heasley LE, Bunn PA Jr, et al (1999) Synergistic effects of new chemopreventive agents and conventional cytotoxic agents against human lung cancer cell lines. Cancer Res 59: 6178–6184. 10626810

[16] Dohadwala M, Batra RK, Luo J, Lin Y, Krysan K, et al (2002) Autocrine/paracrine prostaglandin E2 production by non-small cell lung cancer cells regulates matrix metalloproteinase-2 and CD44 in cyclooxygenase-2-dependent invasion. J Biol Chem 277: 50828–50833. 12393872 10.1074/jbc.M210707200 PMC1471886

[17] Dohadwala M, Yang SC, Luo J, Sharma S, Batra RK, et al (2006) Cyclooxygenase-2-Dependent Regulation of E-Cadherin: Prostaglandin E2 Induces Transcriptional Repressors ZEB1 and Snail in Non-Small Cell Lung Cancer. Cancer Res 66: 5338–5345. 16707460 10.1158/0008-5472.CAN-05-3635

[18] Katoh H, Hosono K, Ito Y, Suzuki T, Ogawa Y, et al (2010) COX-2 and prostaglandin EP3/EP4 signaling regulate the tumor stromal proangiogenic microenvironment via CXCL12-CXCR4 chemokine systems. Am J Pathol 176: 1469–1483. 20110411 10.2353/ajpath.2010.090607 PMC2832166

[19] Sharma S, Yang SC, Zhu L, Reckamp K, Gardner B, et al (2005) Tumor cyclooxygenase-2/prostaglandin E2-dependent promotion of FOXP3 expression and CD4+ CD25+ T regulatory cell activities in lung cancer. Cancer Res 65: 5211–5220. 15958566 10.1158/0008-5472.CAN-05-0141

[20] Li H, Sorenson AL, Poczobutt J, Amin J, Joyal T, et al (2011) Activation of PPARgamma in myeloid cells promotes lung cancer progression and metastasis. PLoS One 6: e28133. 22145026 10.1371/journal.pone.0028133 PMC3228753

[21] Weiser-Evans MC, Wang XQ, Amin J, Van Putten V, Choudhary R, et al (2009) Depletion of cytosolic phospholipase A2 in bone marrow-derived macrophages protects against lung cancer progression and metastasis. Cancer Res 69: 1733–1738. 19208832 10.1158/0008-5472.CAN-08-3766 PMC2653105

[22] Norris PC, Reichart D, Dumlao DS, Glass CK, Dennis EA (2011) Specificity of eicosanoid production depends on the TLR-4-stimulated macrophage phenotype. J Leukoc Biol 90: 563–574. 21653236 10.1189/jlb.0311153 PMC3157897

[23] Bonventre JV, Huang Z, Taheri MR, O'Leary E, Li E, et al (1997) Reduced fertility and postischaemic brain injury in mice deficient in cytosolic phospholipase A2. Nature 390: 622–625. 9403693 10.1038/37635

[24] Gijon MA, Zarini S, Murphy RC (2007) Biosynthesis of eicosanoids and transcellular metabolism of leukotrienes in murine bone marrow cells. J Lipid Res 48: 716–725. 17179116 10.1194/jlr.M600508-JLR200

[25] Hall LM, Murphy RC (1998) Electrospray mass spectrometric analysis of 5-hydroperoxy and 5-hydroxyeicosatetraenoic acids generated by lipid peroxidation of red blood cell ghost phospholipids. J Am Soc Mass Spectrom 9: 527–532. 9879367 10.1016/S1044-0305(98)00013-0

[26] Duan M, Li WC, Vlahos R, Maxwell MJ, Anderson GP, et al (2012) Distinct macrophage subpopulations characterize acute infection and chronic inflammatory lung disease. J Immunol 189: 946–955. 22689883 10.4049/jimmunol.1200660

[27] Janssen WJ, Barthel L, Muldrow A, Oberley-Deegan RE, Kearns MT, et al (2011) Fas determines differential fates of resident and recruited macrophages during resolution of acute lung injury. Am J Respir Crit Care Med 184: 547–560. 21471090 10.1164/rccm.201011-1891OC PMC3175550

[28] Gautier EL, Shay T, Miller J, Greter M, Jakubzick C, et al (2012) Gene-expression profiles and transcriptional regulatory pathways that underlie the identity and diversity of mouse tissue macrophages. Nat Immunol 13: 1118–1128. 23023392 10.1038/ni.2419 PMC3558276

[29] Nomura DK, Hudak CS, Ward AM, Burston JJ, Issa RS, et al (2008) Monoacylglycerol lipase regulates 2-arachidonoylglycerol action and arachidonic acid levels. Bioorg Med Chem Lett 18: 5875–5878. 18752948 10.1016/j.bmcl.2008.08.007 PMC2593629

[30] Horn L, Backlund M, Johnson DH (2009) Targeting the eicosanoid pathway in non-small-cell lung cancer. Expert Opin Ther Targets 13: 675–688. 19409031 10.1517/14728220902915567 PMC2891200

[31] Singh RK, Gupta S, Dastidar S, Ray A (2010) Cysteinyl leukotrienes and their receptors: molecular and functional characteristics. Pharmacology 85: 336–349. 20516735 10.1159/000312669

[32] Kenny PA, Lee GY, Bissell MJ (2007) Targeting the tumor microenvironment. Front Biosci 12: 3468–3474. 17485314 10.2741/2327 PMC2841020

[33] Condeelis J, Pollard JW (2006) Macrophages: obligate partners for tumor cell migration, invasion, and metastasis. Cell 124: 263–266. 16439202 10.1016/j.cell.2006.01.007

[34] Joyce JA, Pollard JW (2009) Microenvironmental regulation of metastasis. Nat Rev Cancer 9: 239–252. 19279573 10.1038/nrc2618 PMC3251309

[35] Nomura DK, Morrison BE, Blankman JL, Long JZ, Kinsey SG, et al (2011) Endocannabinoid hydrolysis generates brain prostaglandins that promote neuroinflammation. Science 334: 809–813. 22021672 10.1126/science.1209200 PMC3249428

[36] Gronich JH, Konieczkowski M, Gelb MH, Nemenoff RA, J.R S (1994) Interleukin-1a causes rapid activation of cytosolic phospholipase A2 by phosphorylation in rat mesangial cells. J Clin Invest 93: 1224–1233. 8132762 10.1172/JCI117076 PMC294074

[37] Nemenoff RA, Winitz S, Qian N-X, Van Putten V, Johnson GL, et al (1993) Phosphorylation and activation of a high molecular weight form of phospholipase A2 by p42 MAP kinase and protein kinase C. J Biol Chem. 268: 1960–1964. 8380583

